# The anti-tumor activities of Neferine on cell invasion and oxaliplatin sensitivity regulated by EMT *via* Snail signaling in hepatocellular carcinoma

**DOI:** 10.1038/srep41616

**Published:** 2017-01-30

**Authors:** Ganlu Deng, Shan Zeng, Junli Ma, Yan Zhang, Yanling Qu, Ying Han, Ling Yin, Changjing Cai, Cao Guo, Hong Shen

**Affiliations:** 1 Institute of Medical Sciences, Xiangya Hospital, Central South University, Changsha, Hunan, 410008, China; 2 Department of Oncology, Xiangya Hospital, Central South University, Changsha, Hunan, 410008, China; 3 Key Laboratory for Molecular Radiation Oncology of Hunan Province, Xiangya Hospital, Central South University, Changsha, Hunan, 410008, China

## Abstract

Tumor invasion and chemotherapy resistance, which are associated with epithelial-mesenchymal transition (EMT), remain as major challenges in hepatocellular carcinoma (HCC) treatment. Neferine, a natural component of Nelumbo nucifera, have been proven the antitumor efficiency in cancer, but the effects of Neferine on HCC invasion and chemosensitivity need to be elucidated. Applying multiple assays of cell proliferation, flow cytometry, immunofluorescence staining, qRT-PCR, Western blot, fluorescence molecular tomography imaging, the influences of Neferine on EMT-regulated viability, apoptosis, invasion, and oxaliplatin (OXA) sensitivity were assessed in HCC cells of HepG2 and Bel-7402, as well as in xenograft animal models *in vivo*. Here, we reported that Neferine had no obvious effects on HCC cells proliferation, but significantly enhanced cytotoxicity and apoptosis caused by OXA *in vitro* and *in vivo*. Through an upregulation of E-cadherin and downregulation of Vimentin, Snail and N-cadherin, Neferine suppressed EMT-induced migration and invasion abilities of HCC cells. TGF-β1 cancelled the effects of Neferine on the migration and invasion of HCC cells. Snail overexpression or TGF-β1-induced EMT attenuated Neferine-mediated OXA sensitization in HCC. Together, our data suggest that Neferine enhances oxaliplatin sensitivity through an inhibition of EMT in HCC cells. Neferine may be used as an OXA sensitizer in HCC chemotherapy.

Hepatocellular carcinoma (HCC) is the second most common cause of death worldwide^1^. Surgical resection is an effective treatment for hepatocellular carcinoma (HCC) at an early stage. A large population of HCC patients is diagnosed with advanced disease and oxaliplatin (OXA)-based chemotherapy is considered to be an important treatment choice for advanced-stage HCC. However, recurrence and metastasis after surgery, as well as poor chemosensitivity persist as a major barrier to successful chemotherapy in HCC.

Epithelial-mesenchymal transition (EMT) is a process characterized by the loss of typical epithelial characteristics and the acquisition of mesenchymal traits^2^. Undergoing EMT progress, HCC cells lose the connection of cell-cell, the contact of cell-matrix, and normal epithelial polarity while gaining mesenchymal characteristics to migrate and invade the surrounding matrix^3^. Reorganization of the extracellular matrix (ECM) is a key event in the process of EMT and malignant cell invasion. The matrix metalloproteases (MMPs) are major components of the enzyme cascade responsible for ECM degradation, remodeling of cell-cell and cell-matrix interactions. MMPs activities are involved in many disease-related processes including cancer progression, invasion and metastasis^4^.

During EMT, expressions of epithelial cell markers like E-cadherin are downregulated, while the mesenchymal cell markers, such as Vimentin and N-cadherin, are upregulated^5^. Recently accumulated evidences indicate that EMT contributes to chemoresistance and the non-EMT cells are more sensitive to chemotherapy in cancers^6^,^7^. Our previous study found that HCC cells with EMT phenotype acquired OXA-resistance^8^. Since that, EMT suppression may be synergistic with conventional chemotherapy to improve HCC prognosis.

Neferine (CAS No.: 2292-16-2) is a major bisbenzylisoquinoline alkaloid derived from the seed embryo of lotus (Nelumbo nucifera, a traditional medicinal plant), demonstrating the nontoxic nature of it^9^. Previous studies had shown that Neferine exerted extensive cardio-protective effects, such as anti-hypertensive, anti-arrhythmic^10^, anti-agglutinating, anti-thrombotic^11^, cholinesterase inhibition^12^, as well as its anti-diabetes functions^13^. Importantly, Neferine was suggested to have antitumor effects and sensitized the cancer cells to chemotherapeutic agents. The multidrug resistance was documented to be reversed in human gastric carcinoma cells by Neferine^14^. Neferine inhibited lung cancer cells growth^15^,^16^ and potentiated anti-cancer effect on lung cancer when combined with doxorubicin^17^. Meanwhile, Neferine could inhibit the proliferation of HCC cells^18^,^19^ and osteosarcoma cells^20^. Neferine was also reported to induce autophagy of ovarian cancer cells^21^. However, the effects of Neferine on HCC invasion and chemosensitivity need to be elucidated.

Based on the existed evidence and understanding, the present study aims to prove our hypothesis that Neferine may have some anti-tumor effects on the invasion and chemosensitivity through EMT regulation in HCC cells.

## Results

### The effects of Neferine on cells viability in HCC and normal hepatic cells

**Figure 1:**
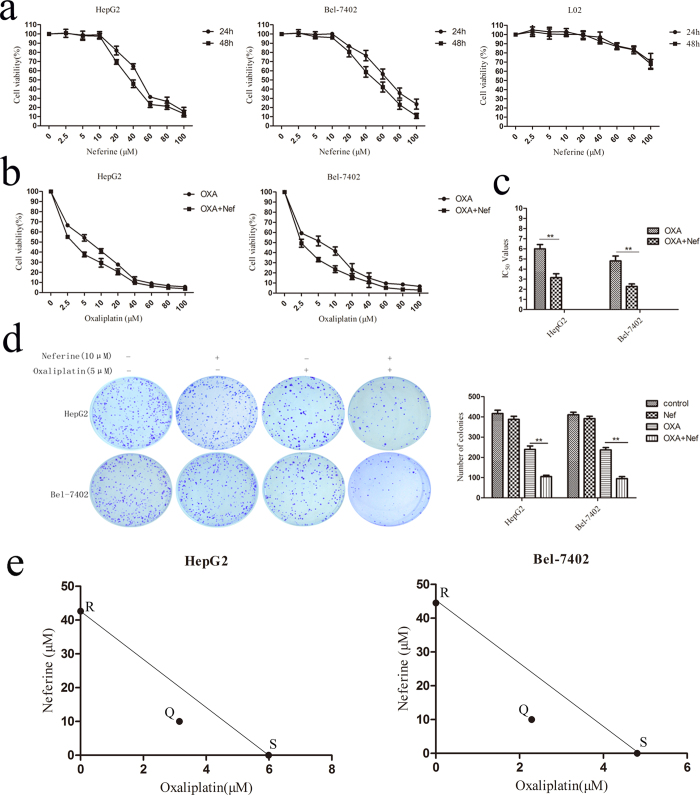
Effects of Neferine on cell proliferation of HCC cells and human normal liver cell lines: (**a**) Cells viability of HepG2, Bel-7402 and L02 treated with different concentrations of Neferine for 24 hrs and 48 hrs were determined by CCK-8. (**b**) HCC cells were co-treated with 10 μM Neferine and different concentrations of OXA for 48 hrs. Neferine promoted OXA sensitivity in HCC. (**c**) Neferine significantly decreased the IC_50_ values of OXA in HCC cells. (**d**) Neferine enhanced OXA-induced cells growth inhibition on colony formation assay in HCC cells. (**e**) Synergism effect between Neferine and OXA quantitatively analyzed with isobologram. Scatter points “S” at the horizontal axis represent the IC_50_ of HCC cells when treated with OXA alone and scatter points “R” at vertical axis represent the IC_50_ of HCC cells when treated with Neferine alone. Scatter points “Q” represent the IC_50_ of OXA when co-treated with Neferine. “Q” points appeared under the straight line the connecting “S” and “R” points, suggesting that synergism effect existed between Neferine and OXA. Nef: Neferine, ^**^p < 0.01.

L02, HepG2 and Bel-7402 cells were treated with Neferine at different concentrations for 24 hrs and 48 hrs. As shown in Fig. 1a, Neferine at the dose up to 10 μM exhibited no significant inhibition of HepG2 cells viability (*p* = 0.7311). HepG2 cells was a little less sensitive to Neferine than Bel-7402, whose viability slightly decreased without statistical difference at 10 μM (*p* = 0.0810). However, Neferine treated at high doses (>10 μM) significantly inhibited cells viability in HepG2 and Bel-7402 cells (*p *< 0.05) in a dose-dependent manner. L02 cells sustained cell viability of over 85% at 80 μM of Neferine, showing no cytotoxic effects of Neferine on normal liver cells. Therefore, a dose of 10 μM Neferine nontoxic to HCC cells was chosen for combined treatment with OXA in further experiments.

### OXA-inhibited HCC growth was potentiated by Neferine

The dose-dependent viability of HCC cells was firstly measured in the group of OXA treatment alone for 48 hrs to determine its IC_50_, which was 5.99 ± 0.35 μM for HepG2 and 4.81 ± 0.39 μM for Bel-7402. OXA at 5 μM, which was the nearest concentration to the IC_50_ values of OXA treatment alone, was then chosen for further co-treatment combined with Neferine.

The cells were subsequently treated with OXA at different concentrations combined with Neferine at 10 μM for 48 hrs. Neferine significantly improved OXA-induced cytotoxicity in HepG2 and Bel-7402 cells. As compared with the control group without any treatment, 5 μM OXA induced 45.91 ± 1.86% growth inhibitions in HepG2, which was considerably increased to 62.35 ± 1.35% in co-treatment group of OXA + Neferine (*p* < 0.01). In Bel-7402 cells, the growth inhibition rate in OXA treatment group was significantly improved in the combined treatment group of 5 μM OXA and 10 μM Neferine (47.23 ± 3.27% *vs.* 64.22 ± 2.86%, *p* < 0.05, Fig. 1b). Meanwhile, Neferine obviously reduced the IC_50_ values of OXA from 5.99 ± 0.35 μM to 3.15 ± 0.32 μM for HepG2, from 4.81 ± 0.39 μM to 2.29 ± 0.20 μM for Bel-7402 cells (Fig. 1c, *p* < 0.01, respectively). In colony formation assays, the group of OXA + Neferine had a significantly decreased colony amounts than the OXA treatment group (*p* < 0.01, Fig. 1d). These results indicated that Neferine significantly increased the chemosensitivity of HCC cells to OXA, implying a synergistic effect between OXA and Neferine. To quantitatively analyze the interactions between Neferine and OXA, an arithmetic method of isobologram indicated a synergistic effect between Neferine and OXA (Fig. 1e).

### Neferine promoted OXA-induced apoptosis of HCC cells

**Figure 2:**
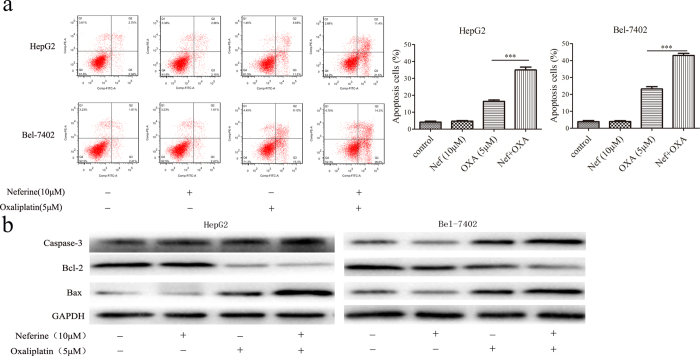
(**a**) Neferine enhanced OXA-induced apoptosis of HCC cells (OXA *vs.* OXA + Neferine, *p* < 0.001, respectively). Apoptosis rate in HCC treated with 10 μM Neferine and/or 5 μM OXA for 48 hrs were detected with flow cytometry by Annexin V/PI staining. **(b)** 10 μM Neferine downregulated anti-apoptotic protein of Bcl-2, but upregulated apoptotic molecular of caspase 3 and Bax in 5 μM OXA-treated HCC cells for 48 hrs. ^***^p < 0.001.

The flow cytometry results exhibited that Neferine at the concentration of 10 μM did not have significant apoptosis induction ability. In Neferine treatment and control groups, the apoptosis rates were 4.16% ± 0.33 and 4.75% ± 0.28 for HepG2 cells (*p* = 0.21), 3.90% ± 0.41 and 4.0% ± 0.31 (*p* = 0.87) for Bel-7402 cells, respectively. The apoptosis rates of HepG2 cells were 16.42% ± 0.48 induced by OXA and 34.97 ± 1.0% induced by OXA combined with Neferine (OXA *vs.* OXA + Neferine, *p* < 0.001). Consistently, combination regimens showed higher apoptosis rate of Bel-7402 cells as compared with single OXA agent (23.24 ± 0.76% *vs.* 43.0 ± 0.72%, OXA *vs.* OXA + Neferine, *p* < 0.001, Fig. 2a). Western blot revealed that the expression of anti-apoptotic protein Bcl-2 was decreased significantly but that of pro-apoptotic proteins Bax and active caspase-3 was increased significantly in the co-treatment group, as compared with that in individual agent (either OXA or Neferine) treatment group (Fig. 2b).

### Establishment of TGF-β1-induced EMT model in HCC cells

**Figure 3:**
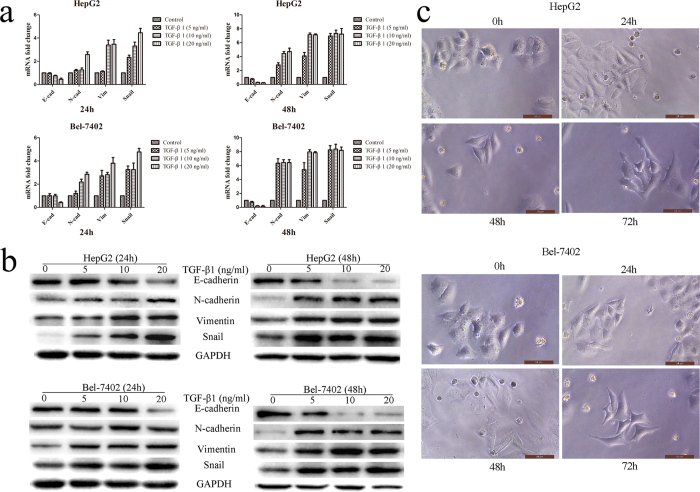
TGF-β1 induced EMT progress and restored migration, invasion abilities suppressed by Neferine. **(a,b)** TGF-β1 induced EMT progress. HCC cells were treated with 5 ng/ml, 10 ng/ml, 20 ng/ml TGF-β1 for 24 hrs or 48 hrs to induce EMT. mRNA and protein expression of EMT biomarkers (E-cadherin, N-cadherin & Vimentin), and EMT promoting transcription factor (Snail) was determined by qRT-PCR and by Western blot. Original blots of high-contrast blots are presented in Supplementary Fig. S4. **(c)** Morphological changes of HCC cells treated with 10 ng/ml TGF-β1 for different times. TGF-β1 changed cells morphology from pebble-like epithelial to dispersed, spindle-like mesenchymal and pseudopodium stretching. (Original magnification: ×400).

Since EMT played an important role in invasion and chemoresistance of cancer cells, we explored whether EMT and invasion capacity were regulated by Neferine in HCC cells. We found that Neferine alone inhibited cells migration/invasion abilities (Supplementary Fig. S1, *p* < 0.01, respectively), and promoted HCC epithelial cells HepG2 and Bel-7402 to more epithelial phenotype by increasing E-cadherine expression and decreasing Vimentin/N-cadherin/Snail expression (Supplementary Fig. S2). We next established a model of TGF-β1-induced EMT in HCC cells in time- and dose-dependent manners to further investigate the association between EMT progress and Neferine-promoted chemosensitivity of HCC cell. HepG2 cells and Bel-7402 cells were treated with different concentrations (5 ng/ml, 10 ng/ml, 20 ng/ml) of TGF-β1 for 24 hrs and 48 hrs. qRT-PCR and Western blot revealed that TGF-β1 at the dose of 10 ng/ml for 48 hrs induced obvious EMT of HCC cells with decreased expression level of E-cadherin but increased expression level of N-cadherin and Vimentin as compared with the untreated cells (Fig. 3a,b). The expression of Snail, a transcription factor of EMT, rapidly increased at 24 hrs with the administration of TGF-β1 (Fig. 3a,b). Being stimulated with 10 ng/ml TGF-β1 for 48 hrs, cells gradually elongated and dispersed. The administration of TGF-β1 changed cells morphology from pebble-like epithelial to spindle-like mesenchymal and pseudopodium stretching after 72 hrs (Fig. 3c). Taken together, 10 ng/ml TGF-β1 for 48 hrs was chosen to induce HCC EMT in further experiments.

### Neferine reversed TGF-β1-induced EMT phenotype in HCC cells

**Figure 4:**
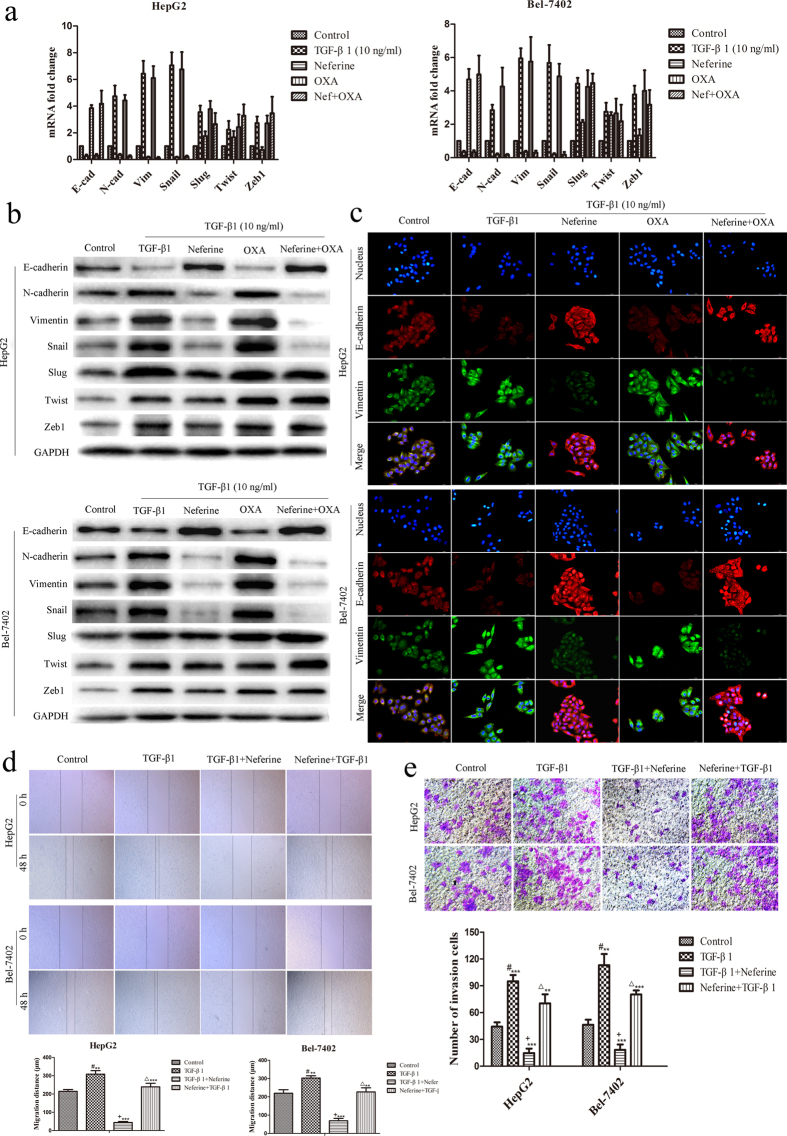
The reversion effects of Neferine on TGF-β1-induced EMT model in HCC cells. **(a,b)** HCC cells were firstly treated with TGF-β1 for 48 hrs to induce EMT before the administration of Neferine (48 hrs) and/or OXA (48 hrs). mRNA and protein expression of epithelial marker (E-cadherin), mesenchymal markers (N-cadherin & Vimentin), and EMT promoting transcription factor (Snail, Slug, Twist, Zeb1), which was determined by qRT-PCR and by Western blot in TGF-β1-treated HCC cells treated with Neferine and/or OXA. Original blots of high-contrast blots are presented in Supplementary Fig. S5. **(c)** Representative double immunofluorescence staining for expression and co-localization of E-cadherin and Vimentin in TGF-β1-treated HCC cells treated with Neferine and/or OXA. (Original magnification: ×400). **(d)** HCC cells treated with 10 ng/ml TGF-β1 (TGF-β1 groups) or co-treated with 10 ng/ml TGF-β1 and Neferine (TGF-β1 + Neferine groups: applied Neferine to TGF-β1-treated HCC cells for 48 hrs). In Neferine + TGF-β1 groups, HCC cells were pre-treated with Neferine for 48 hrs before the administration of TGF-β1). Migration abilities of HepG2 and Bel-7402 cells were inhibited by Neferine *in vitro* and were canceled by TGF-β1 in wound healing assays. #: TGF-β1 *vs.* control, *p *< 0.01, respectively; + : TGF-β1 *vs.* TGF-β1 + Neferine, *p *< 0.001, respectively; Δ: TGF-β1 + Neferine *vs.* Neferine + TGF-β1, *p *< 0.001, respectively. **(e)** Invasion abilities of HepG2 and Bel-7402 cells examined by transwell assays were inhibited by Neferine *in vitro* and were canceled by TGF-β1. TGF-β1 groups: treated HCC cells with 10 ng/ml TGF-β1 for 48 hrs; TGF-β1 + Neferine groups: applied Neferine to TGF-β1-treated HCC cells for 48 hrs; Neferine + TGF-β1 groups: pre-treated HCC cells with Neferine for 48 hrs before the administration of TGF-β1. #: TGF-β1 *vs.* control, *p *< 0.01, respectively; + : TGF-β1 *vs.* TGF-β1 + Neferine, *p *< 0.001, respectively; Δ: TGF-β1 + Neferine *vs.* Neferine + TGF-β1, *p *< 0.01, respectively. ^**^p < 0.01, ^***^p < 0.001.

Due to the important roles of EMT in invasion and chemoresistance of cancer cells, we further explored whether EMT was regulated by Neferine in HCC cells. qRT-PCR, Western blot and immunofluorescence staining were respectively applied to detect the expression levels and co-localization of EMT biomarkers in Neferine and/or OXA-treated HCC cells with the administration of 10 ng/ml TGF-β1. TGF-β1 induced EMT phenotype as compared to the control groups, as well as the same phenomenon occurred in TGF-β1 co-treatment with OXA groups. Comparing to the TGF-β1 groups, Neferine treatment alone and combined with OXA groups resulted in a down-regulated mRNA and protein expression of Vimentin and N-cadherin, concomitant with an up-regulated expression of E-cadherin in TGF-β1-treated HCC cells. Moreover, mRNA and protein expression of transcription factor Snail, which is critical for EMT induction, was significantly decreased in Neferine treatment and co-treatment with OXA groups. Expression of Slug, another EMT transcription factor, was decreased in Neferine-treated HepG2 but not in Bel-7402 cells. The expression of the other EMT-related transcription factors of Twist and Zeb1 was not significantly regulated by Neferine treatment (Fig. 4a,b). Cellular immunofluorescence labeled by E-cadherin and Vimentin antibodies confirmed the suppression effects of Neferine on TGF-β1-induced EMT in HepG2 and Bel-7402 cells (Fig. 4c).

### Neferine inhibited TGF-β1-promoted migration and invasion abilities of HCC cells

EMT is involved in many aspects of cellular behaviors, such as the mobility of cancer cells. Herein, we found the suppression function of Neferine on TGF-β1-induced EMT, so that we further explored the effects of Neferine on migration and invasion abilities of TGF-β1-treated HCC cells. Wound healing assays showed that the migration ability of HCC cells was significantly promoted by TGF-β1 treatment (Fig. 4d, Control *vs.* TGF-β1, *p* < 0.01, respectively) but repressed by Neferine treatment in TGF-β1-treated HCC cells (Fig. 4d, TGF-β1 + Neferine *vs.* TGF-β1*, p* < 0.001, respectively). In the results of transwell tests, TGF-β1 groups enhanced obviously invasion ability as compared with untreated control groups (Fig. 4e, Control *vs.* TGF-β1, *p* < 0.01, respectively) while Neferine treatment in TGF-β1-treated HCC cells displayed a significantly lower invasion capacity than the groups consisting of TGF-β1-treated HepG2 and Bel-7402 cells (Fig. 4e, TGF-β1 + Neferine *vs.* TGF-β1, *p* < 0.001, respectively). To confirm the EMT regulation of Neferine, the HCC cells were pre-treated with Neferine for 48 hrs (Neferine + TGF-β1 groups) before the administration of TGF-β1 in wound healing and transwell invasion assays. Interestingly, treatment with TGF-β1 cancelled the suppressed migration and invasion abilities of Neferine-pretreated HCC cells (Fig. 4d,e, Neferine + TGF-β1 *vs.* TGF-β1 + Neferine, *p* < 0.01, respectively).

### Knockdown of Snail inhibited EMT phenotype and migration, but enhanced OXA sensitivity

**Figure 5:**
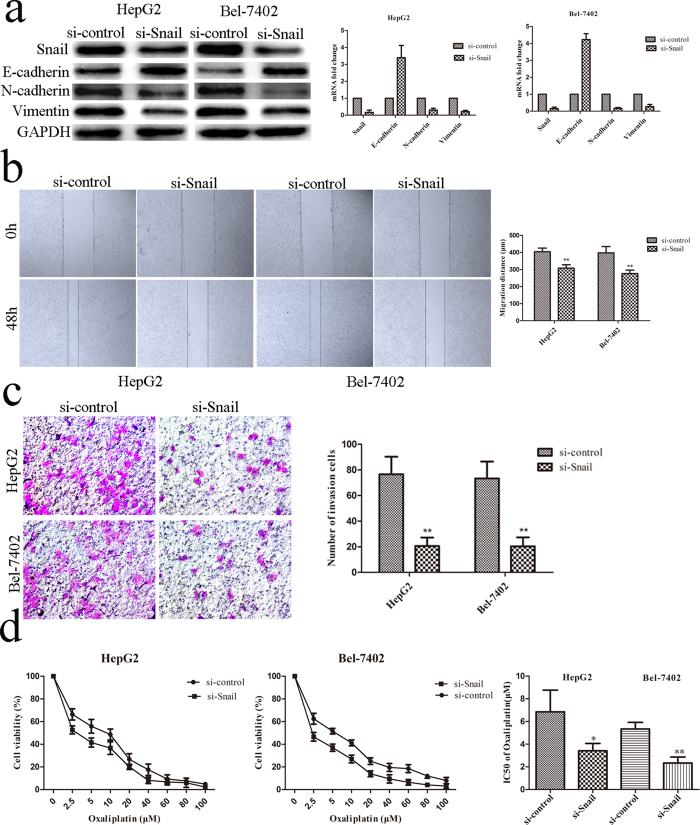
Knockdown of Snail inhibited EMT progress, migration/invasion capacities and enhanced OXA sensitivity. **(a)** qRT-PCR and Western blot confirmed decreased expression level of Snail in si-Snail groups. Knockdown of Snail caused the changes of EMT biomarkers with E-cadherin upregulation and downregulation of N-cadherin/Vimentin. **(b,c)** Knockdown of Snail inhibited migration and invasion capacities of HCC in wound healing assays and transwell invasion assays. si-control *vs.* si-Snail, *p *< 0.01, respectively. **(d)** Knockdown of Snail increased OXA sensitivity by CCK-8 assays. Cells were treated with different concentrations of OXA for 48 hrs. IC_50_ of OXA: si-control *vs.* si-Snail, *p *< 0.05, respectively. ^*^p < 0.05, ^**^p < 0.01, ^***^p < 0.001.

To determine the roles of Snail in HCC cells’ migration and chemosensitivity, we knocked down the expression of Snail with siRNA in HCC cells and performed further study. The expression changes of EMT markers in HepG2 and Bel-7402 cells were assayed by qRT-PCR and Western blot. Comparing to the si-control groups, Snail knockdown significantly decreased the expression levels of mesenchymal markers of N-cadherin and Vimentin, and increased the expression levels of epithelial marker of E-cadherin (Fig. 5a). Wound healing and transwell assays indicated that knockdown of Snail significantly inhibited HCC cells’ migration and invasion abilities as compared to the si-control groups (Fig. 5b,c. *p* < 0.01, respectively). Si-Snail groups enhanced the sensitivity of HCC cells against OXA as compared with the si-control groups by decreasing the IC_50_ values of OXA from 6.844 ± 1.108 μM to 3.411 ± 0.375 μM for HepG2 and from 5.339 ± 0.338 μM to 2.340 ± 0.304 μM for Bel-7402 (Fig. 5d, *p* < 0.05, respectively), implying the correlation of Snail and chemosensitivity.

### Snail overexpression induced EMT and eliminated migration suppression and OXA sensitivity regulated by Neferine in HCC cells

**Figure 6:**
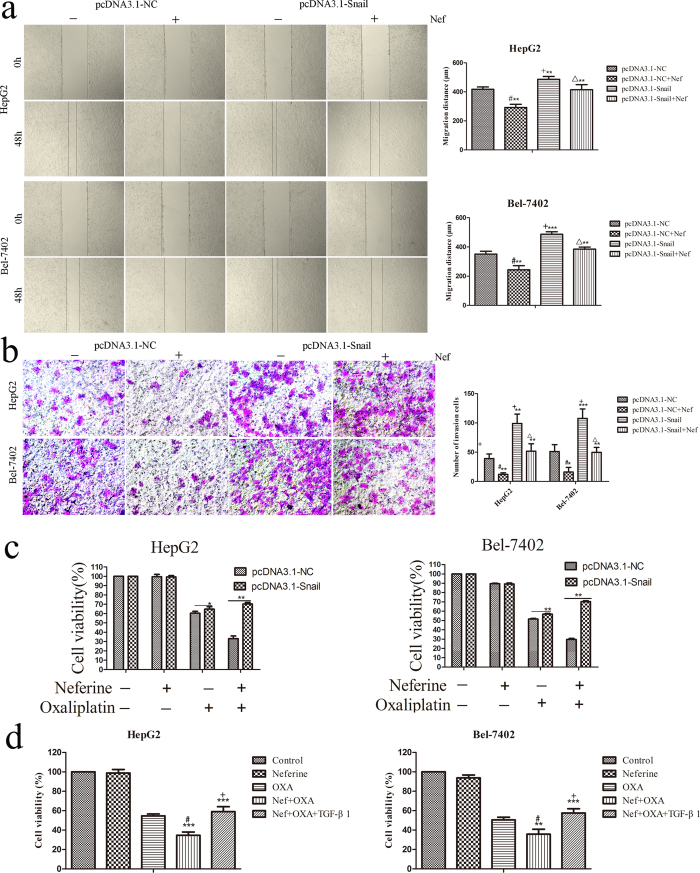
Snail overexpression restored cells migration and invasion abilities attenuated OXA sensitized by Neferine treatment. **(a,b)** Migration and invasion abilities suppressed by Neferine treatment were restored by Snail overexpression. HepG2 and Bel-7402 cells were pre-treated with or without 10 μM Neferine and then transfected by pcDNA3.1-Snail and pcDNA3.1-NC vectors for 48 hrs, respectively. #: pcDNA3.1-NC + Nef *vs.* pcDNA3.1-NC, *p *< 0.05, respectively; + : pcDNA3.1-Snail *vs.* pcDNA3.1-NC, *p *< 0.01, respectively; Δ: pcDNA3.1-Snail + Nef *vs.* pcDNA3.1-Snail, *p *< 0.01, respectively. **(c)** OXA sensitization by Neferine treatment was eliminated by Snail overexpression in HepG2 and Bel-7402 cells. **(d)** OXA sensitization by Neferine treatment was eliminated by 10 ng/ml TGF-β1 in HepG2 and Bel-7402 cells. #: Nef + OXA *vs.* OXA, *p *< 0.01, respectively; +: Nef + OXA + TGF-β1 *vs.* Nef + OXA, *p *< 0.001, respectively. ^*^p < 0.05, ^**^p < 0.01, ^***^p < 0.001.

The above results exhibited the suppression of Snail expression by Neferine and the association of Snail with migration and chemosensitivity in HCC cells. We further investigated the functions of Snail overexpression on EMT, Neferine**-**suppressed migration and Neferine-promoted OXA sensitivity in HCC cells. Snail overexpression by Snail plasmid (pcDNA3.1-Snail) transfection induced an obvious EMT progress, which was recognized by decreased E-cadherin expression and increased Vimentin/N-cadherin expression (Supplementary Figure S3a–c). Neferine treatment caused obvious migration and invasion suppression in the blank vector transfection groups (Fig. 6a,b, pcDNA3.1-NC *vs.* pcDNA3.1-NC + Neferine, *p *< 0.05, respectively). Consequently, EMT induced by Snail overexpression significantly enhanced the migratory and invasive capabilities of Snail-transfected HCC cells as compared to pcDNA3.1-NC groups (Fig. 6a,b, *p* < 0.001, respectively). Migratory and invasive capabilities were significantly enhanced in pcDNA3.1-Snail groups which were pre-treated with Neferine for 48 hrs than the control groups transfected with the blank vector (pcDNA3.1-NC) which were pre-treated with Neferine (Fig. 6a,b, *p* < 0.001, respectively), implying that overexpression of Snail eliminated migration suppression by Neferine. Based on the chemoresistance induced by EMT, Snail transfection significantly attenuated OXA sensitization by Neferine in HepG2 and Bel-7402 cells. CCK-8 assays indicated that pcDNA3.1-Snail groups had significantly decreased OXA sensitivity than pcDNA3.1-NC groups (Fig. 6c, *p* < 0.01, respectively).

### TGF-β1 attenuated OXA sensitization by Neferine

To further confirm the roles of EMT regulation in chemosensitivity by Neferine, we examined cells viability treated with Neferine and OXA and TGF-β1 by CCK-8 assays. TGF-β1 groups exhibited higher cells viability than the groups with combined treatment of Neferine and OXA without TGF-β1, suggesting TGF-β1 attenuated OXA sensitization by Neferine (Fig. 6d, *p* < 0.001, respectively).

### Neferine increased OXA sensitivity *via* EMT inhibition in xenograft nude mice

**Figure 7:**
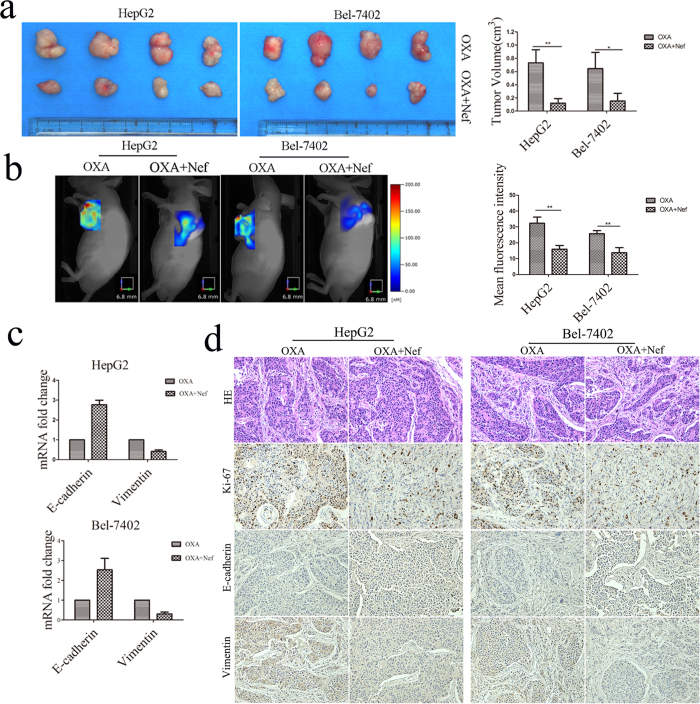
Neferine suppressed EMT so as to increased OXA sensitivity in xenograft nude mice. **(a)** The size of subcutaneous tumors in OXA and OXA + Neferine treatments were calculated by formula (mm^3^) = (L × W^2^) × 0.5. Tumor volumes: OXA + Nef *vs.* OXA, *p* < 0.05, respectively. **(b)** MMPs activities in the subcutaneous orthotopic tumors. Applying a Fluorescence Molecular Tomography imaging system, the mean fluorescence intensities were imaged and calculated as total MMPs fluorescence signal/fluorescence volume to represent MMPs activities. Mean fluorescence intensity: OXA + Nef *vs.* OXA, *p* < 0.01, respectively. **(c)** Neferine inhibited HCC EMT *in vivo*. mRNA expression of EMT biomarkers in HCC tumor was assessed by qRT-PCR**. (d)** HE and IHC staining of Ki-67, E-cadherin and Vimentin in mice tumors (×200 magnification) indicated that Neferine depressed EMT and increased OXA sensitivity *in vivo*. ^*^p < 0.05, ^**^p < 0.01.

We established subcutaneous xenograft tumor models to verify the effects of Neferine on EMT-regulated OXA sensitivity of HCC *in vivo*. As shown in Fig. 7a, HepG2 cell-derived tumors at the implantation sites treated with OXA alone were statistically larger than that treated with OXA and Neferine (0.73 ± 0.01 cm^3^ *vs.* 0.12 ± 0.03 cm^3^, *p *< 0.01). Consistently, Bel-7402 cell-derived tumors in OXA treatment group grew more rapidly than that in OXA/Neferine co-treatment group (0.64 ± 0.12 cm^3^ *vs.* 0.15 ± 0.05 cm^3^, *p *< 0.05).

The mean MMPs activities, which were involved in EMT and represented by the fluorescence intensities, in OXA treatment groups were significantly higher than that in OXA/Neferine co-treatment groups (HepG2: 32.31 ± 2.27 *vs.* 15.95 ± 1.37, *p *< 0.01; Bel-7402: 25.72 ± 1.13 *vs.* 13.79 ± 1.83, *p *< 0.01, Fig. 7b). Expression of cell proliferation index (Ki-67) and EMT biomarkers of E-cadherin/Vimentin, which was detected by qRT-PCR and IHC, indicated that Neferine treatment significantly depressed HCC EMT phenotype and cell proliferation in xenograft tumor (Fig. 7c,d).

## Discussion

Over years, a great deal of efforts has been taken to increase chemosensitivity in HCC patients. However, owing to little and limited understanding of chemoresistant mechanisms, the prognosis of HCC remains poor. New therapeutic strategies which potentiate chemotherapeutic sensitivity safely provide a promising approach for effective HCC treatment. The present study indicated that co-administration of Neferine, a natural component of Nelumbo nucifera, could enhance OXA chemosensitivity in HCC.

Regarding to the anti-tumor effects of Neferine against HCC, Paramasivan *et al*.^18^ reported that Neferine at the dose of 10 μM exhibited obviously cytotoxicity by inducing reactive oxygen species mediated intrinsic pathway of HepG2 apoptosis. However, we did not find any significant effect of Neferine at the concentration of 10 μM on the proliferation of both HepG2 and Bel-7402 cells. Consistent to our finding, Yoon^19^ provided evidences that Neferine at the concentration of 20 μM exhibited significant cytotoxicity against Hep3B cells via ER stress and autophagy induction, while 10 μM Neferine showed no distinct effect. Even at the dose of 20 μM, Neferine, which was extracted by the researchers themselves, did not affect the growth of Sk-Hep1 cells and primary normal hepatocytes of THLE-3. Different liver (cancer) cells may have diverse tolerance to Neferine. Meanwhile, the purity decided by extraction methods may have significant influences on the stability and antitumor activity of Neferine.

In general, a variety of mechanisms contributes to drug resistance, which occurs in almost all chemotherapeutic agents and results in low response and tumor refractoriness in HCC treatment. Accumulated evidences indicate that EMT is associated with proliferation, invasion/migration, metastasis, and chemoresistance in human cancer^22^,^23^. EMT is a dynamic process which can be triggered by many molecules including transforming growth factor-β (TGF-β), Notch, Wnt, tumor necrosis factor-α (TNF-α), and other cytokines^24^,^25^. MMPs contribute prominently to their signals in micro-environment *via* degrading structural components of ECM and promote invadopodia formation to activate EMT process, permitting tumor invasion and metastasis^26^,^27^,^28^. Indeed, EMT plays an important role in invasion and chemoresistance associated at molecular and phenotypic levels^7^,^29^,^30^. It was reported that tumor cells acquired EMT phenotype reduced susceptibility to chemotherapy by increasing apoptotic resistance, upregulation of chemoresistance and drug metabolizing genes including drug transporters aldehyde dehydrogenases (ALDHs), cytochrome P450s, and glutathione-metabolism-related enzymes^6^. EMT induced by transcription factor of Snail was found to attenuate cell cycle through blocking the G1/S transition due to downregulation of Cyclin D2 transcription^31^. Under this condition, decreased activities of caspase 3/8 in Snail-expressing cells and high activities of MEK/Erk and PI3K/Akt signaling led to the upregulation of pro-apoptosis Bcl-2 family^32^. Thus, Snail conferred resistance to cell death, suggesting that EMT-induction is prone to resistance to apoptosis. It has also been observed that restoring E-cadherin expression increased sensitivity to epidermal growth factor receptor inhibitors in lung cancer cells, while mesenchymal-like cells were resistance to drug treatment^33^. Meanwhile, Snail suppressed TGF-β-induced apoptosis and was sufficient to trigger EMT in hepatocytes^34^. EMT inhibition could be a useful strategy to cause a loss of anti-apoptotic signal and/or trigger apoptotic responses to sensitize cancer cells to chemotherapy. Accompanying with caspase-3 activation, upregulating of Bax and downregulating Bcl-2 expression, Neferine exerted a promotion of OXA-induced HCC apoptosis through EMT inhibition in this study.

Moreover, EMT-generated properties of cancer stem cells (CSCs) are important reasons contributing to chemoresistance in human cancers^35^,^36^. EMT facilitates the generation of CSCs with the mesenchymal traits which are required for dissemination and chemoresistance^37^. The cells with CSC phenotype (CD44high, CD24low) in breast cancer were found to be resistant to neoadjuvant chemotherapy^38^. Furthermore, Snail was associated with CSCs-like traits acquisition and mediated cell survival in ovarian cancer effectively^39^. PI3K pathway, which was confirmed to be activated in Snail-expressing cells^31^, was directly linked to CSCs expansion and maintenance via promoting the proliferation of CSCs in breast cancer^40^ and prostate cancer^41^. Targeting CSCs therapy therapeutically seems to overcome drug resistance. Antibiotic salinomycin could kill breast CSCs preferentially and induced the differentiation of mesenchymal-like cancers *in vivo*, as assessed by increased E-cadherin expression and decreased vimentin expression^42^. We demonstrated that Snail overexpression induced EMT and eliminated OXA sensitization effects of Neferine in HCC. However, the roles of EMT-elicited HCC CSCs in OXA sensitivity promoted by Neferine needs to be further clarified.

In conclusion, Neferine significantly suppressed EMT so as to inhibit cell mobility but increased OXA sensitivity *via* Snail signaling in HCC. Our findings suggest that Neferine may be a potent OXA sensitizer in HCC to improve the patients’ chemotherapy response.

## Methods

### Chemicals

Neferine and oxaliplatin were purchased from Sigma-Aldrich Corp. (St. Louis, MO). TGF-β1 were purchased from PeproTech (Rocky Hill, NJ). The chemical agents were dissolved and stored in accordance with the manufacture’s protocol.

### Cell culture

HCC cells HepG2 and Bel-7402, and human normal liver cell line L02, were obtained from the Cell Bank of Typical Culture Preservation Committee of Chinese Academy of Science, Shanghai, China. The cells were cultured in Dulbecco’s modified Eagle’s medium (DMEM) supplemented with 10% fetal bovine serum (FBS), 100 U/mL penicillin sodium and 100 μg/mL streptomycin sulfate (Gibco, Grand Island, NY) at 37 °C under an atmosphere of 95% air and 5% CO_2_.

### Cell proliferation and clonogenic assay

Cells were seeded on a 96-well plate at a density of 5.0 × 10^3^/well overnight and then were subjected to various concentrations of OXA with or without Neferine for 48 hrs. Cell viability was assayed by Cell Counting Kit-8 (CCK-8, Dojindo Molecular Technologies, Inc., Tokyo, Japan). Briefly, added 10 μL CCK-8 to each well and the absorbance at 450 nm was measured. The wells absent of drugs were used as the controls. The 50% inhibitory concentration (IC_50_) was calculated from the survival curves. Each assay was performed in triplicate. An arithmetic method of isobologram was employed to quantitatively analyze the effect of drug synergism^43^.

For colony formation assays, cells were seeded in 60-mm dishes at a density of 1 × 10^3^/dish. Add OXA and/or Neferine after 24 hrs and then cultured for 2 weeks to form colonies. Fixed the cells with methanol and stained with 0.1% crystal violet for 20 mins.

### Apoptosis assay

A FACS Canto II flow cytometer (BD Biosciences, San Jose, CA) was used to quantitate the apoptosis rate by Annexin V-FITC Apoptosis Detection Kit (BD Biosciences). HCC cells in different groups were harvested and suspended in 100 μL binding buffer. Added 5 μL of Annexin V-FITC and 5 μL of propidium iodide (PI) for 15 mins in darkness. 100 μL binding buffer was added and the cells’ apoptosis were detected in 1 hr.

### Cell migration and invasion assays

#### Wound healing assay

Cells were seeded in 6-well plate for 24 hrs. The confluent cell monolayers were scratched by a 200 μL pipette tip straightly. The cells were washed with PBS for 2–3 times and subsequently cultured in fresh medium with 2% FBS for 48 hrs. The wound healing of the scratched cells were photographed under a DMIL LED AE2000 inverted microscope (Leica, Wetzlar, Germany).

#### Transwell invasion assay

The invasion abilities of HCC cells were assessed using Matrigel-coated upper inserts contained polycarbonate filters in 8-μm pore size (BD Biosciences). Culture medium containing 10% FBS was placed in the lower chambers to act as a chemoattractant. 4 × 10^4^ cells suspended in 200 μL serum-free DMEM were seeded in the upper chambers and incubated at 37 °C for 48 hrs. The cells that penetrated the Matrigel-coated filter were stained with 0.1% crystal violet hydrate solution.

### Immunofluorescence staining

Fixed treated cells with 4% paraformaldehyde for 25 mins and permeabilized with 0.1% Triton X-100 for 10 mins. Blocked with 2% bovine serum albumin (BSA) for 30 mins at 37 °C and followed by the primary antibody against E-cadherin (1:200, Abcam, Cambridge, UK) and Vimentin (1:500, Abcam) at 4 °C overnight. The cells were subsequently incubated with the corresponding Alexa Fluor 546 or Alexa 488-conjugated secondary antibody for 1 hr at room temperature. The nuclei were stained with DAPI for 3 mins. The images were captured using a DMI-4000B inverted fluorescence microscopy (Leica).

### Quantitative real-time reverse transcription polymerase chain reaction (qRT-PCR)

Total RNA was isolated using Trizol Reagent (Invitrogen) and the cDNA was synthetized using the PrimeScript™ Kit (TaKaRa Bio Inc., Otsu, Japan) following the manufacturer’s instructions. qRT-PCR was performed in triplicate using SYBR Green fluorescent-based assay (TaKaRa Bio Inc.) on a ViiA^TM^7 RT-PCR system (Applied Biosystems, Carlsbad, CA). The primers for real-time PCR were listed as follows: E-cadherin Forward: 5′-TACGCCTGGGACTCCACCTA-3′, Reverse: 5′-CCAGAAACGGAGGCCTGAT-3′; Vimentin Forward: 5′-TGTGGATGT TTCCAAGCCTGAC-3′, Reverse: 5′-GAGTGGGTATCAACCAGAGGGAG-3′; N-cadherin Forward: 5′-CCACGCCGAGCCCCAGTATC-3′, Reverse: 5′-CCCCCA GTCGTTCAGGTAATCA-3′; Snail Forward: 5′-CACTATGCCGCGCTCTTTC-3′, Reverse: 5′-GCTGGAAGGTAAACTCl′GGATTAGA-3′; GAPDH Forward: 5′-CGG AGTCAACGGATTTGGTCGTAT-3′, Reverse: 5′-AGCCTTCTCCATGGTGGTGA AGAC-3′; Slug Forward: 5′-GGGCTCAGTTCGTAAAGG-3′, Reverse: 5′-GAGGAGGTGTCAGATGGA-3′; Twist Forward: 5′-TTTACATCCGATTTACTGC-3′, Reverse: 5′-CCTAATGCTTTCCCTCAT-3′; Zeb1 Forward: 5′-AAGTGGCGGTAGATGGTA-3′, Reverse: 5′-TTGTAGCGACTGGATTTT-3′. Relative mRNA expression levels were calculated by the 2^−ΔCt^ [ΔCt = Ct (targeting gene)-Ct (GAPDH)] method and were normalized to the internal control of GAPDH.

### Western blot

Total protein was extracted in RIPA lysis buffer, separated by SDS-PAGE and then transferred onto PVDF membrane (Millipore, Bedford, MA). The membranes were blocked in TBST containing 5% skim milk at 37 °C for 2 hrs and respectively incubated with the primary antibodies at 4 °C overnight against Bcl-2 (1:200), Bax (1:1000), active caspase-3 (1:200), Vimentin (1:1000), E-cadherin (1:5000), Snail (1:500), Slug (1:1000), Twist (1:500), Zeb1 (1:1000), GAPDH (1:5000, Abcam), and N-cadherin (1:1000, Cell Signaling Technologies, Beverly, MA), followed by an incubation with HRP-conjugated secondary antibody (1:5000, Abcam) for 1 hr at 37 °C. Employing the enhanced chemiluminescence kit (Millipore), the bands were automatically visualized using a ChemiDoc XRS + system (Bio-Rad, Hercules, CA) and quantitatively analyzed with Image Lab software (Bio-Rad). GAPDH expression was used as the internal control to normalize the sample loading amounts.

### Xenograft model

To investigate the effects of Neferine on OXA sensitivity *in vivo*, a xenograft animal model was constructed in male BALB/c mice (4-week old). Briefly, 5 × 10^6^ HepG2 and Bel-7402 cells were suspended in 150 μL serum-free DMEM and injected subcutaneously into the left flank regions. One week later, the mice received an intraperitoneal injection of OXA (0.8 mg/kg/w) with or without Neferine (20 mg/kg/d) for 3 weeks. MMPSense™ 750 FAST fluorescent imaging agent (PerkinElmer, Boston, MA) was injected intravenously and the mice were imaged 6 hrs later using a FMT-4000 3D Fluorescence Molecular Tomography imaging system (PerkinElmer). The mean fluorescence intensity reflecting MMPs activities in tumor tissues was equal to the total MMPs fluorescence signal/fluorescence volume, which was captured and calculated by the Quantitative Tomography *in vivo* Imaging Software of TrueQuant ™ (PerkinElmer). Tumor volumes were calculated using formula volume = (length × width^2^) × 0.5. All animal studies were conducted in the Animal Institute of Central South University according to the protocols approved by the Medical Experimental Animal Care Commission of the University.

### Immunohistochemistry (IHC)

The HCC tissues in the xenograft tumors were fixed in 10% formalin, dehydrated, and embedded in paraffin. 5 μm-thick sections were then stained against Ki67 (1:200, Abcam), E-cadherin (1:200, Abcam) and Vimentin (1:400, Abcam).

### siRNA transfection

Two candidate small interfering RNA (siRNA) sequences targeting human Snail gene (GenBank Accession No. NC_000020.11) were designed as follows (RiboBio, Guangzhou, China): siRNA1: sense 5′-CCUUCGUCCUUCUCCUCUAdTdT-3′, antisense 5′-dTdTGGAAGCAGGAAGAGGAGAU-3′; siRNA2: sense 5′-ACUCAGAUGUCAAGAAGUAdTdT-3′, antisense 5′-dTdTUGAGUCUACAGUUCUUCAU-3′, respectively. The control siRNA was synthesized and purified by RiboBio (Guangzhou, China). HepG2 and Bel-7402 cells were transfected with 10 nM (final concentration) Snail siRNA and control siRNA using Lipofectamine 2000 (Invitrogen) according to manufacturer’s protocols. Downregulated expression of Snai1 was confirmed by qRT-PCR and Western blot in triplicate.

### Transient transfections of Snail cDNA

To explore the roles of Snail in Neferine-regulated EMT in HCC cells, 1 × 10^5^ cells seeded into 6-well plates were transiently transfected with a pcDNA3.1 expression vector containing a full-length of human Snail sequence (pcDNA3.1-Snail) using Lipofectamine-2000 (Invitrogen) in accordance with the manufacture’s protocol. HCC cells were transfected in parallel with the corresponding blank vector as the control (pcDNA3.1-NC). 48 hrs after the transfection, cells with different treatment were collected for qRT-PCR and Western blot analyses.

### Statistical analysis

All experiments were performed in triplicate. Statistical analysis was performed using SPSS software (Version 17.0, SPSS Inc, Chicago, IL). All data were presented as mean ± SD and analyzed by Student’s t-test or one-way ANOVA. *p* < 0.*05* was considered to be statistically significant.

## Additional Information

**How to cite this article:** Deng, G. *et al*. The anti-tumor activities of Neferine on cell invasion and oxaliplatin sensitivity regulated by EMT *via* Snail signaling in hepatocellular carcinoma. *Sci. Rep.* **7**, 41616; doi: 10.1038/srep41616 (2017).

**Publisher's note:** Springer Nature remains neutral with regard to jurisdictional claims in published maps and institutional affiliations.

## Supplementary Material

Supplementary Information
