# Supplementary material for: The anti-tumor activities of Neferine on cell invasion and oxaliplatin sensitivity regulated by EMT via Snail signaling in hepatocellular carcinoma
Source: Sci Rep. 2017 Jan 30;7:41616. doi: 10.1038/srep41616 (PMC5278559; doi:10.1038/srep41616)
Supplement: Supplementary Information [file srep41616-s1.pdf]

The anti-tumor activities of Neferine on cell invasion and oxaliplatin sensitivity regulated by EMT via Snail signaling in hepatocellular carcinoma

Author name: Ganlu Deng [1, 2], Shan Zeng [1, 2, 3], Junli Ma [2, 3], Yan Zhang [1, 2], Yanling Qu[1, 2], Ying Han [1, 2], Ling Yin [1, 2], Changjing Cai [1, 2], Cao Guo [1, 3], Hong Shen [1, 2, 3 *].

Author's affiliation: 1 Institute of Medical Sciences, 2 Department of Oncology, 3 Key Laboratory for Molecular Radiation Oncology of Hunan Province, Xiangya Hospital, Central South University, Changsha, Hunan, China 410008

* Address reprint requests to: Dr. Hong Shen,

Institute of Medical Sciences, Xiangya Hospital,

Central South University, Changsha, Hunan, China 410008.

Tel: (86) 731-8432-7633

E-mail: hongshen2000@csu.edu.cn

Supplementary Information includes:
Supplementary Figure S1-S5.

Figure S1

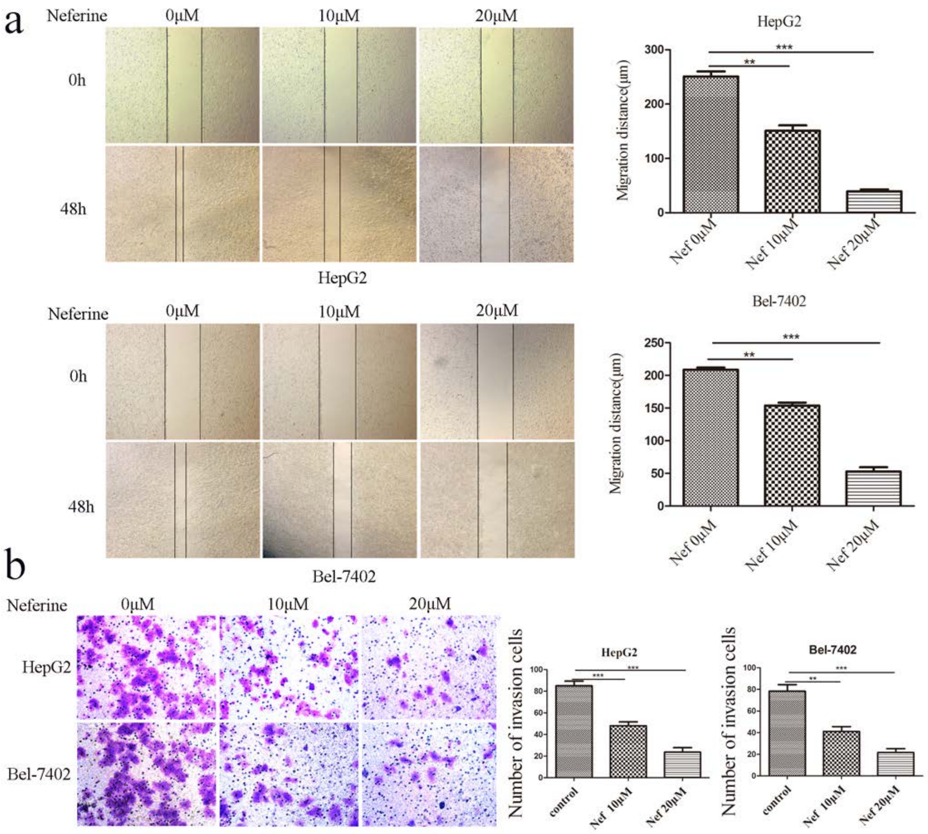

Figure S1: HCC cells migration, invasion ability inhibited by Neferine alone in vitro. (a) Migration ability tested by wound healing assay in HepG2 and Bel-7402 cells treated with Neferine at different concentrations. (b) Invasion ability examined by transwell assay in HepG2 and Bel-7402 cells treated with Neferine. *: $p < 0.05$, **: $p < 0.01$, ***: $p < 0.001$.

Figure S2

Figure S2: The suppression effects of Neferine on EMT in HCC cells. (a & b) mRNA and protein expression of epithelial marker (E-cadherin), mesenchymal markers (N-cadherin & Vimentin), and EMT promoting transcription factor (Snail), which was determined by qRT-PCR and by Western blot in HCC cells treated with Neferine at different concentrations. (c) Representative double immunofluorescence staining for expression and co-localization of E-cadherin and Vimentin in HCC (Original magnification: ×400).

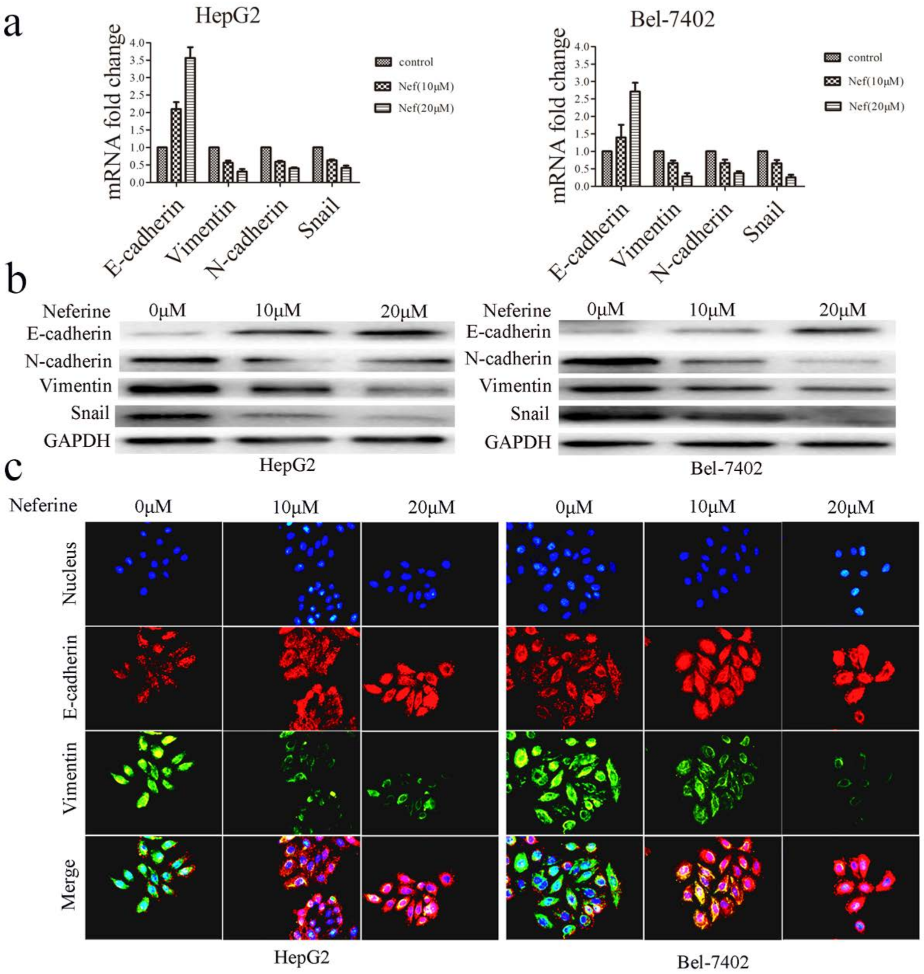

Figure S3

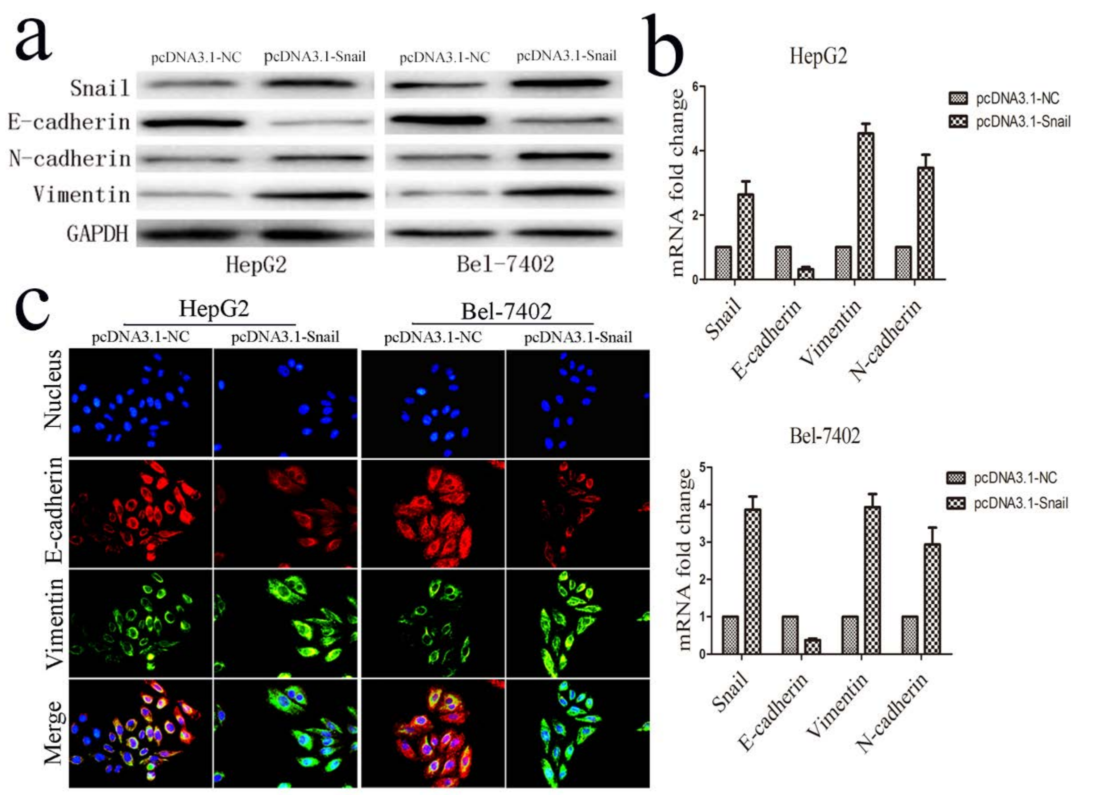

Figure S3: Snail overexpression promoted HCC EMT. (a & b) HCC EMT phenotype enhanced by Snail overexpression via increasing expression of mesenchymal markers and decreasing expression of epithelial marker. HepG2 and Bel-7402 cells were transfected by pcDNA3.1-Snail and pcDNA3.1-NC vectors for 48 hrs, respectively. (c) Expression and co-localization of E-cadherin and Vimentin protein in HepG2 and Bel-7402 cells regulated by overexpressed Snail, which was imaged by double immunofluorescence staining (Original magnification: ×400).

Figure S4

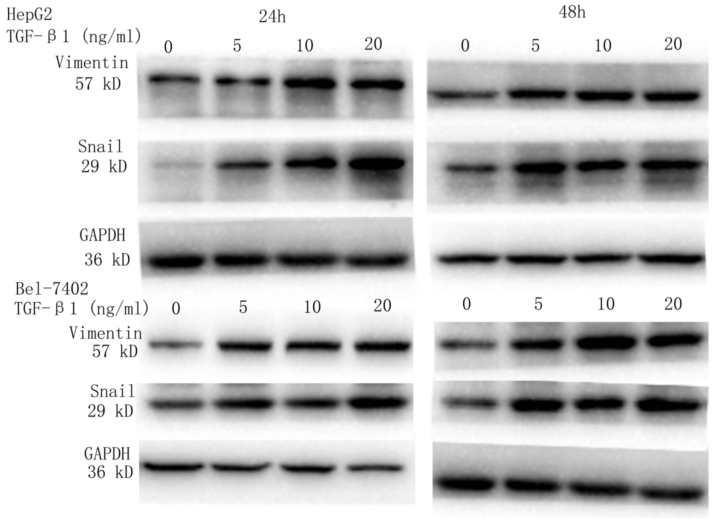

Figure S4: Original blots of high-contrast blots of Figure 3b. TGF-β1 induced EMT progress.HCC cells were treated with 5 ng/ml, 10 ng/ml, 20 ng/ml TGF-β1 for 24 hrs or 48 hrs to induce EMT. EMT biomarkers and EMT promoting transcription factor was determined by Western blot.

Figure S5

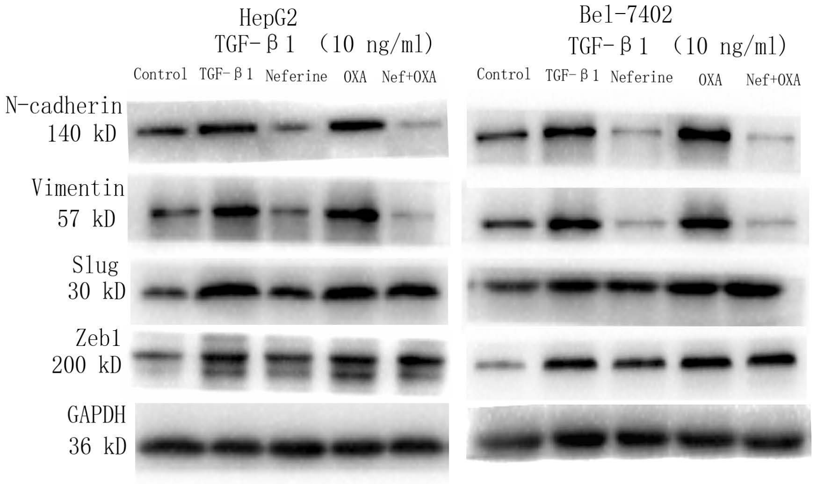

Figure S5: Original blots of high-contrast blots of Figure 4b. The suppression effects of Neferine on TGF-β1-induced EMT in HCC cells. Protein expression of EMT markers and EMT promoting transcription factors were determined Western blot in TGF-β1-induced HCC cells treated with Neferine and/or OXA.
